# Attentional Biases toward Attractive Alternatives and Rivals: Mechanisms Involved in Relationship Maintenance among Chinese Women

**DOI:** 10.1371/journal.pone.0136662

**Published:** 2015-08-26

**Authors:** Yidan Ma, Guang Zhao, Shen Tu, Yong Zheng

**Affiliations:** 1 Key Laboratory of Cognition and Personality (MOE), Southwest University, Chongqing, China; 2 Research Center of Psychological Development and Education, Liaoning Normal University, Dalian, China; 3 Department of Psychology, China West Normal University, Nanchong, China; Zhejiang Key Laborotory for Research in Assesment of Cognitive Impairments, CHINA

## Abstract

A long-term romantic relationship can offer many benefits to committed individuals. Thus, humans possess relationship maintenance mechanisms to protect against threats from those who serve as attractive alternatives or intrasexual rivals. Many studies have indicated that romantic love can act as a commitment device to activate these mechanisms. To examine the attentional bias associated with relationship maintenance among 108 college students (49 single and 59 committed females) in China, we used a semantic priming procedure to activate mental representations associated with romantic love and then asked participants to complete a dot-probe task for the purpose of making a distinction between the engage and disengage components of attention. No significant engaging effects toward attractive faces were observed among committed females, but the following significant disengaging effects were found: when primed with romantic love, single females showed increased attention toward and difficulty in disengaging from attractive male faces, whereas females already in a committed relationship did not alter their attention, remaining as inattentive to attractive alternatives as they were in the baseline condition. In addition, committed females responded to love priming by exhibiting difficulty in disengaging from attractive rivals. The present findings provide evidence in the Chinese cultural context for the existence of early-stage attentional processes in the domain of relationship maintenance that committed Chinese females protected an ongoing relationship by not only being inattentive to attractive males who could serve as attractive alternatives, but also being more attentive to attractive females who could be potential rivals when mental representations associated with romantic love were primed.

## Introduction

A committed long-term romantic relationship can facilitate bi-parental care and increase the chances of offspring survival, which is especially important to a woman [1], and satisfies one’s fundamental need for positive social bonds [2]. Moreover, a steady and satisfying romantic relationship usually offers positive psychological and health outcomes [2, 3]. However, one of the major threats to the stability of a heterosexual relationship is the temptation of attractive opposite-sex persons [4, 5], who also serve as intrasexual rivals for one’s partner. The availability of attractive alternatives can threaten one’s satisfaction with, investment in, and commitment to the current relationship, and is a predictor of relationship breakup [6–8]. Consequently, the threat from attractive alternatives or intrasexual rivals can lead to destructive consequences for both members of a romantic partnership. However, psychological mechanisms that can help people maintain their long-term relationships may not only occur in connection with higher-order social cognition (e.g., evaluations, choices), such as the derogation effect, whereby people involved in a committed relationship tend to devalue the physical attractiveness of attractive others [9–11], but also involve relatively automatic, lower-order stages of perceptual processes (e.g., attention, memory) [12–16].

Indeed, researchers using a visual cuing task to assess biases in attentional disengagement have identified at least two separate psychological mechanisms involving early-stage visual processing, which may aid in relationship maintenance. These mechanisms are as follows [14–18]: attentional adhesion to (or difficulty in disengaging from) one’s intrasexual rivals or inattention to one’s attractive alternatives. First, attentional adhesion to attractive same-sex others facilitates the identification of potential intrasexual threats that could help guard one’s mate from those threats [14]. Maner, Gailliot, and DeWall [14] asked participants to identify a probe (a circle or square) correctly and as quickly as possible, with each probe following a target picture of an attractive or average face that appeared for 500 ms in either the same location as the face (filler trials) or a different quadrant (incongruent trials). The reaction times (RTs) for incongruent trials reflected the disengage component of attention, and it was found that committed females who felt insecure about their relationships took longer to disengage from attractive same-sex targets than did committed and secure women. Similarly, Maner et al. [15] found that priming with jealousy led committed individuals who were more concerned with threats posed by potential intrasexual rivals, to increase their attentional adhesion to attractive same-sex targets. Second, inattention to attractive alternatives could help committed individuals resist the allure of those alternatives and remain committed to one’s current partner [7, 19]. Maner, Rouby, and Gonzaga [16] used a love-priming procedure and asked participants to write a brief essay about a romantic experience with their current partner before completing a visual cuing task, in order to examine committed individuals’ attentional bias toward attractive alternatives when feelings of romantic love were elicited. Compared to the control condition, in which participants wrote a brief essay about a happy time, love priming led to decreased attentional adhesion of committed participants toward attractive opposite-sex targets, but had no significant effect on attention to average opposite-sex targets, average same-sex targets, or attractive same-sex targets. Furthermore, Maner, Gailliot, and Miller [17] found that relationship motives could inhibit the effects of implicit mating priming on attentional adhesion to attractive alternatives, observing that committed individuals were less attentive than single individuals were to attractive opposite-sex targets in the mating-priming condition.

In the current study, we explored the relationship maintenance mechanisms involved in lower-order perceptual processing among Chinese women, because maintenance of a long-term relationship seems to be important for committed females within the Chinese cultural context [20, 21]. On the one hand, traditional Chinese culture places strict moral constraints on a woman’s chastity and loyalty to her husband [21]. This is in line with the Chinese saying about “being faithful to one’s husband until death.” On the other hand, owing to the polygamous nature of the ancient marriage structure that was common in China, men’s infidelity is still tolerated in modern Chinese society [22, 23]. Because of the maintenance of a traditionally male-dominated culture, women still take a subordinate role in the marriage relationship [24]. This can lead to committed females losing their material resources when a long-term relationship ends. Thus, we expected that committed Chinese females would have similar relationship maintenance mechanisms at the early stages of visual processing.

According to previous studies, automatic attentional biases associated with relationship maintenance might not take effect unless participants experience mating-related motivational states [15, 16]. From an evolutionary perspective, the motives having the most immediate impact on the perception of other people are likely to be those that, over the course of human evolutionary history, have been closely linked to differential reproductive success [18]. Thus, in the current study, we used a semantic priming procedure to activate a sample of Chinese females’ mental representations associated with romantic love, in order to activate mating-related motives, and examined the extent to which participants’ attention was automatically held by images of same-sex and opposite-sex targets that varied in levels of facial attractiveness. Our study varies in a number of ways from previous studies in the area, notably compared with Maner et al. [16] (see Table 1). First, we investigated whether love priming would lead single vs. committed females to alter their early-stage attention toward attractive opposite-sex targets. On the one hand, feelings of romantic love could motivate single individuals to seek an attractive partner who appears to have high genetic fitness [25–27]. On the other hand, feelings of romantic love for committed individuals can act as a commitment device that helps people reduce their interest in attractive alternatives [16, 28]. Thus, we predicted that love priming would increase difficulty with disengagement from attractive opposite-sex targets among single females, but not among females already in a committed romantic relationship. Second, we explored whether committed females differed in terms of the early-stage attention paid to attractive same-sex targets when primed with romantic love. Experimentally eliciting feelings of love can trigger committed individuals who are predisposed toward jealousy to feel concern about partner infidelity [29], leading to difficulties in disengaging from attractive rivals [15, 18]. Some studies have suggested that Chinese students, especially female students, tend to associate love with jealousy and betrayal [30, 31]. Thus, we predicted that love priming would increase attentional difficulties with disengaging from attractive same-sex targets among committed females.

**Table 1:** Comparison of methods and findings used by Maner, Rouby, and Gonzaga (2008) and those used in the current study.

	Maner, Rouby, and Gonzaga (2008)	The current study
Participants	Committed males and females from a Western culture	Different: Single and committed females from an Eastern culture
		Same: Nothing
Priming procedure	Scenario priming—participants wrote a brief essay about a time they experienced strong feelings of romantic love for their current partner	Same: Love priming
		Different: Semantic priming—each love word was displayed to participants for 500 ms
Stimuli materials	Attractive males, Attractive females, Average males, Average females	Same: Same facial types
		Different: Each facial stimulus was presented with a neutral stimulus, including attractive male-N pairs, attractive female-N pairs, average male-N pairs, average female-N pairs, and N-N pairs
Experimental paradigm	Visual cuing task to assess biases in attentional disengagement (modified dot-probe task)	Same: Dot-probe task
		Different: Modified dot-probe task to assess the engage and disengage components of attention
Attention component	Engagement was not examined	Same: Engaging attention with the target face
		Different: Measured by RTs for congruence trials compared to RTs for N-N trials. Engaging index = RT_N–N_ − RT_congruence_
	Disengagement was measured by RTs for incongruence trials	Same: Attention shift away from the location of the target face to a different location
		Different: Measured by RTs for incongruence trials compared to RTs for N-N trials. Disengaging index = RT_incongruence_ − RT_N–N_
Findings	Attentional disengagement: Committed participants in the love-priming condition were significantly less attentive than committed participants in the control condition were to attractive opposite-sex targets	Same: Committed females in the love-priming condition were inattentive to attractive opposite-sex targets.
		Different: Committed females in the love-priming condition did not alter their attention, remaining as inattentive to attractive alternatives as they were in the baseline condition. They were significantly less attentive than single females were to attractive opposite-sex targets in the love-priming condition.
		Different: Committed females in the love-priming condition increased attention toward and difficulty in disengaging from attractive same-sex targets.

N, neutral picture; RTs, reaction times.

In addition, in the domain of attentional bias associated with relationship maintenance, most studies have focused on the disengage component of attention and have indicated that this can play a role in relationship maintenance (see Introduction, paragraph 2). However, there are at least two subsystems in the human attention system, comprising engaging attention with the stimulus and disengaging attention from the stimulus [32]. Enhanced attentional engagement toward a threat could be related to improved awareness of that threat in one’s environment, and possibly be aimed at facilitating conscious threat appraisal [33]. Attractive opposite- and same-sex individuals both serve as relationship threats to committed individuals. Quick attentional engagement with attractive faces in a complex social environment facilitates the identification of potential threats from other members, which could help protect a current relationship. Thus, in this study, to probe whether the engage component of attention that could be used to help relationship maintenance also occurred among committed females in the context of attractive individuals, we used a modified dot-probe task that allowed for differentiation between the engage and disengage components of attention by adding trials with only neutral stimuli [34, 35].

In the current study, we examined the relationship maintenance mechanisms involved in early-stage visual processing among Chinese women. We predicted that there would be interactions among love priming, levels of facial attractiveness, and participants’ relationship status, such that love priming would increase attentional difficulty with disengaging from attractive opposite-sex targets among single females, but not among committed females, and increase difficulty with disengagement from attractive same-sex targets among committed females. In addition, we probed whether the engage component of attention was activated toward attractive individuals among committed females, for the presumed purpose of relationship maintenance. We hope to provide support for existing theories of relationship maintenance in Eastern cultures.

## Method

### Participants

One-hundred and eight female undergraduates (age range = 18–24 years) were recruited from Southwest University by Internet advertisement and each was paid RMB 20 (about USD 3.2). All were heterosexual, right-handed, and had normal or corrected-to-normal vision. Of the participants, 49 were single (mean age = 21.16 years, *SD* = 1.39) and 59 were currently in a committed romantic relationship (mean age = 21.39 years, *SD* = 1.44). All committed individuals were in an exclusive relationship and the mean relationship length was 19.02 months (*SD* = 12.19, range = 3–54 months). The committed participants completed the commitment subscale of the Companionate Love Scale [36], which comprises four items on a scale from 1 (not at all true) to 9 (definitely true) to measure commitment (α = .75). A sample item is “I have confidence in the stability of my relationship with my boyfriend.” On average, these participants were moderately or highly committed to their current partner (*M* = 7.04, *SD* = 1.22).

### Materials

#### Priming words

We gave an open-ended survey to 30 female undergraduates (mean age = 20.30 years, *SD* = 1.15; none of whom was involved in other parts of this study) for the purpose of generating words associated with love. After analyzing the frequencies of the resulting words, 20 love words (e.g., love letter; 情书in Chinese) were collected. Another 36 female undergraduates (mean age = 20.22 years, *SD* = 1.55; none of whom was involved in other parts of this study) were asked to rate those love words in terms of the extent to which they were associated with love (1 = *not at all* to 7 = *completely*; *M* = 6.12, *SD* = 0.22), and in relation to three emotional information dimensions of pleasure (1 = *very displeasing* to 9 = *very pleasing*; *M* = 7.07, *SD* = 0.64), excitement (1 = *very calm* to 9 = *very exciting*; *M* = 5.37, *SD* = 0.78), and familiarity (1 = *very unfamiliar* to 9 = *very familiar*; *M* = 8.28, *SD* = 0.37). Moreover, 20 control words (e.g., excited; 兴奋in Chinese) were taken from the Chinese Affective Words System [37] to match the priming words in terms of pleasure (*M* = 6.89, *SD* = 0.25; *t* (25) = -1.19, *p* = 0.245) and excitement (*M* = 5.57, *SD* = 0.31; *t* (25) = 1.05, *p* = 0.302).

#### Stimuli materials

Photographs of the faces of unfamiliar college-age Chinese people (67 males and 58 females, posing with a neutral expression) were collected from the Internet by searching using the keyword “images” (证件照 in Chinese) or photographed by the experimenters. Faces were edited to grey scale photographs and equated for size, brightness, and contrast using Adobe Photoshop software. Similar to the facial materials used by Anderson et al. [38], all the images were rated on their levels of attractiveness (1 = *very unattractive* to 7 = *very attractive*) by an independent group of 30 undergraduate judges (12 males and 18 females, mean age = 20.73 years, *SD* = 1.55) who were blind to the purpose of this study and did not participate in other parts of this study. Finally, 20 attractive male (*M* = 5.38, *SD* = 0.87), 20 attractive female (*M* = 5.45, *SD* = 0.87), 20 average male (*M* = 3.17, *SD* = 0.62), and 20 average female (*M* = 3.20, *SD* = 0.60) images were employed in the experiment. The ratings of attractive faces were significantly higher than those of average faces, *t* (39) = 34.4, *p* < 0.001. There were no significant differences between the ratings made by males and females of the faces of attractive male, *t* (38) = -0.04, *p* = 0.97; attractive female, *t* (38) = 0.577, *p* = 0.57; average male, *t* (32) = -0.49, *p* = 0.63; and average female, *t* (38) = -0.44, *p* = 0.66. In addition, 120 neutral pictures (e.g., household furniture), selected from those used by Gao et al. [39], were edited to match the facial stimuli in terms of size, brightness, contrast, and color. Of these neutral pictures, 20 were used in the practice trials and the remaining 100 were used in the experimental trials.

### Design and procedure

A 2 (relationship status: single female vs. committed female, between subjects) × 2 (priming condition: love priming vs. control priming, between subjects) × 4 (target type: attractive male vs. attractive female vs. average male vs. average female, within subjects) × 2 (congruence: congruent trials vs. incongruent trials, within subjects) mixed factorial design was employed.

**Fig 1:**
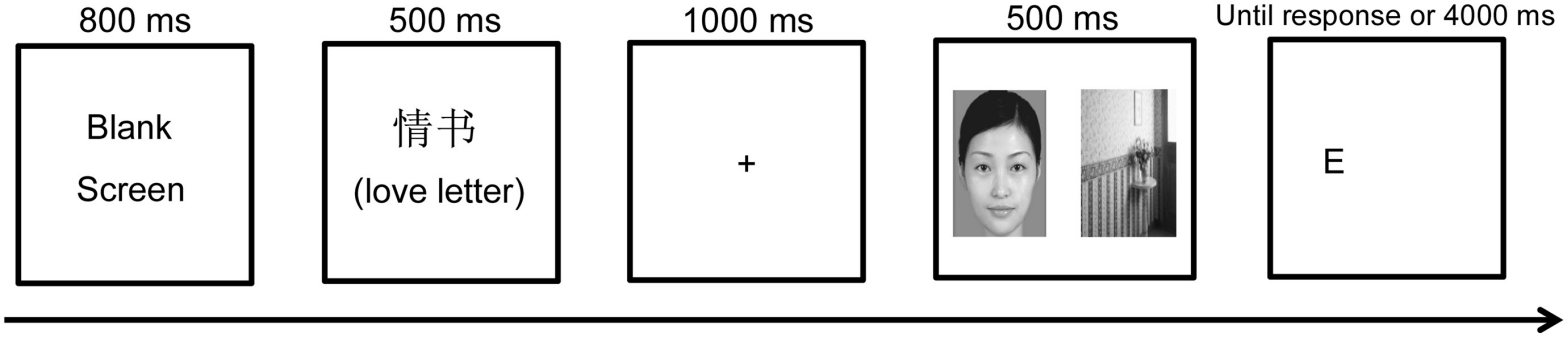
Example of the dot-probe paradigm procedure. This example shows a congruent trial of the attractive female-neutral picture pair in the love-priming condition.

In order to control for the possible priming effects of the love scales, participants completed several items assessing control variables online one day prior to the laboratory session. They reported their relationship length, and completed the Passionate Love Scale [40] (α = .87) and the Companionate Love Scale [36] (α = .80) to assess their love state. Before taking part in the experiment, participants (none of whom was involved in other parts of this study) were informed that the experiment investigated cognitive performance. Participants performed the dot-probe task, programmed with E-Prime software on a desktop computer (19 in) with a refresh rate of 60 Hz and resolution ratio of 1024 × 768, working individually in private laboratory rooms. The computer monitor was placed about 80 cm in front of the participant’s eyes. Each trial began with a blank screen displayed for 800 ms, followed by a priming (or control) word (4.8° × 1.5° visual angle) displayed on the center of the computer screen for 500 ms. In the love-priming condition, participants were primed with love words. In the control (or baseline) condition, participants were primed with control words. After the word disappeared, a black fixation point “+” appeared in the center of the computer screen for 1,000 ms and was then replaced by a pair of pictures (4.1° × 5.1°) that was displayed for 500 ms. The center of each picture was 4.9° lateral to the center of the computer screen. Immediately after the offset of each picture pair, a probe target (“E” or “F,” 0.5° × 1°) was presented at the location of one of the pictures (congruent = probe appeared at the same location as the face; incongruent = probe appeared at the other side of the face; neutral-neutral pairs could not be differentiated as being congruent or incongruent) and participants had to indicate the type of probe by pressing the “1” or “2” key on the keyboard as quickly as they could. The probe was displayed until a response was made, or up to a maximum of 4,000 ms (Fig 1).

Participants completed 10 practice trials and four blocks of 90 experimental trials. Stimuli for the practice trials consisted of neutral picture pairs that were not presented in the experimental trials. In the experimental trials, each block comprised the following five conditions: attractive male-neutral (10 congruent and 10 incongruent trials), attractive female-neutral (10 congruent and 10 incongruent trials), average male-neutral (10 congruent and 10 incongruent trials), average female-neutral (10 congruent and 10 incongruent trials), and neutral-neutral (10 trials). Each picture was presented only once in each block, and each pair was matched in a random order, with no pair repeated. The types and locations of probes were counterbalanced across the experiment. The participants were given a one-minute break between each block. At the end, all participants were carefully probed for suspicion and none recognized the true purpose of the experiment.

Written informed consent was obtained from all participants, and the protocol used in the current study was reviewed and approved by the ethics committee of the School of Psychology at Southwest University, China. In addition, the subject shown in Fig 1 provided written informed consent, as outlined in the PLoS consent form, to publication of her photograph.

### Data preparation

Trials with RTs of less than 200 ms and more than three standard deviations above the sample mean (i.e., 0.23% of trials), and those with errors (i.e., 0.83% of trials) were excluded from the analyses.

### Data analyses

All data analyses were based on the engaging and disengaging indices of RTs, which can be used to control for overall group differences in RTs [41] and directly test of the components of attentional biases [42]. The engaging indices were calculated by subtracting RTs for congruent trials with face-neutral picture pairs from RTs for trials with neutral-neutral picture pairs. A positive score on the engaging index indicates enhanced attentional engagement toward face pictures compared with neutral pictures. The disengaging indices were calculated by subtracting RTs for trials with neutral-neutral picture pairs from RTs for incongruent trials with face-neutral picture pairs. A positive score on the disengaging index is indicative of difficulty with disengagement from face pictures compared with neutral pictures. A zero score denotes no differences in attentional engagement or disengagement for face pictures vs. neutral pictures [35]. According to Linardatos and Lydon [43] and Maner et al. [16], the mean engaging and disengaging indices for average targets (average male and average female) were used to create baseline measures. A 2 (relationship status: single female vs. committed female) × 2 (priming condition: love priming vs. control priming) × 3 (target type: attractive male target vs. attractive female target vs. average target) × 2 (attention component: engage vs. disengage) repeated measures analysis of variance (ANOVA) was performed. Only main effects and interactions relevant to the study’s hypotheses were examined.

In a second set of analyses, we added several control variables to examine whether the effects of interest were robust, even when variables that could influence participants’ attention toward target faces were included. Age was added as a covariate in the analysis among all single and committed participants. Age, logged relationship length, Passionate Love Scale score, and Companionate Love Scale score were added as covariates in the analysis only among committed participants. Relationship length (in months) was log transformed for normalization.

## Results

Means and standard deviations of the RTs for congruent and incongruent trials, and disengaging and engaging indices by target type, priming condition, and relationship status, are presented in Table 2.

**Table 2:** Summary of RT data for single and committed participants in the love-priming and control conditions.

Target type	RT type^a^	Love-priming condition		Control condition	
		Single (*n* = 21)	Committed (*n* = 24)	Single (*n* = 28)	Committed (*n* = 35)
**N**-**N**		584 (61)	557 (81)	540 (58)	562 (60)
**Attractive male**-**N**	Congruent RT	578 (62)	558 (82)	539 (59)	563 (61)
	Engagement	6 (29)	-2 (17)	2 (21)	-1 (13)
**Average male**-**N**	Congruent RT	582 (61)	562 (87)	538 (64)	561 (61)
	Engagement	2 (21)	-6 (17)	2 (23)	1 (18)
**Attractive female**-**N**	Congruent RT	586 (63)	556 (84)	537 (63)	560 (58)
	Engagement	-2 (23)	1 (13)	3 (19)	3 (15)
**Average female**-**N**	Congruent RT	580 (56)	557 (81)	538 (58)	564 (64)
	Engagement	5 (24)	-1 (15)	2 (21)	-2 (21)
**Attractive male**-**N**	Incongruent RT	599 (72)	554 (77)	539 (61)	560 (56)
	Disengagement	14 (21)	-2 (11)	-1 (19)	-1 (18)
**Average male**-**N**	Incongruent RT	582 (59)	554 (76)	541 (61)	563 (65)
	Disengagement	-3 (20)	-3 (20)	1 (23)	1 (19)
**Attractive female**-**N**	Incongruent RT	578 (57)	567 (94)	537 (58)	557 (55)
	Disengagement	-6 (24)	10 (19)	-4 (16)	-5 (20)
**Average female**-**N**	Incongruent RT	582 (46)	559 (86)	541 (60)	559 (60)
	Disengagement	-2 (27)	2 (22)	1 (15)	-3 (17)

RT, reaction time; N, neutral picture.

^a^All types of RT data are reported in ms; standard deviations are shown in parentheses.

### Overall effects

The ANOVA revealed significant interactions among relationship status × priming condition × target type × attention component, *F* (2, 208) = 3.397, *p* = 0.035, partial η^2^ = 0.032. We found the following two other interaction effects that could be subsumed under the significant four-way interaction: relationship status × target type, *F* (2, 208) = 10.378, *p* < 0.001, partial η^2^ = 0.091; target type × relationship status × priming condition, *F* (2, 208) = 9.244, *p* < 0.001, partial η^2^ = 0.082. No other significant effects were observed (all *p*’s > 0.080). When age was added to the analysis as a control variable, the four-way interaction remained significant, *F* (2, 206) = 3.385, *p* = 0.036, partial η^2^ = 0.032. No significant interactions were found for age × target type, *F* (2, 206) = 0.315, *p* = 0.730, partial η^2^ = 0.003; age × attention component, *F* (1, 103) = 0.875, *p* = 0.352, partial η^2^ = 0.008; or age × target type × attention component, *F* (2, 206) = 0.048, *p* = 0.953, partial η^2^ < 0.001. We also conducted a 2 (relationship status: single female vs. committed female) × 2 (priming condition: love priming vs. control priming) × 2 (target type: average male target vs. average female target) × 2 (attention component: engage vs. disengage) repeated measures ANOVA, including age as a covariate, to ensure that there were no differences between the two target types (all *p*’s > 0.127). In order to break down this complex interaction and test the study’s hypotheses, we examined the data for disengage and engage components separately.

### Disengaging index

A 2 × 2 × 3 repeated measures ANOVA of relationship status, priming condition, and target type in relation to the disengaging index revealed significant interaction effects for the target type × relationship status × priming condition, *F* (2, 208) = 10.038, *p* < 0.001, partial η^2^ = 0.088, along with that for target type × relationship status, *F* (2, 208) = 9.150, *p* < 0.001, partial η^2^ = 0.081, and that for target type × priming condition, *F* (2, 208) = 3.090, *p* = 0.048, partial η^2^ = 0.029. No other significant effects were observed (all *p*’s > 0.144).

Further, a 2 (priming condition) × 3 (target type) repeated measures ANOVA was conducted among committed participants. There was a significant target type and priming condition interaction, *F* (2, 114) = 5.578, *p* = 0.005, partial η^2^ = 0.089. No other significant effects were observed (all *p*’s > 0.159). Then, the four covariates of age, logged relationship length, Passionate Love Scale score, and Companionate Love Scale score were included in this analysis. The interaction between target type and priming condition among the committed group remained significant, *F* (2, 106) = 5.289, *p* = 0.006, partial η^2^ = 0.091. None of the interactions between target type and any of the control variables was significant (all *p*’s > 0.101). Further simple effects analyses showed that committed females in the love-priming condition were more attentive to attractive same-sex targets than committed females in the baseline condition, *F* (1, 57) = 8.564, *p* = 0.005, partial η^2^ = 0.131, but no significant effect of priming was observed on attentional biases toward attractive opposite-sex targets among committed females, *F* (1, 57) = 0.043, *p* = 0.836, partial η^2^ = 0.001. Paired-sample *t*-test showed that in the love-priming condition, committed females’ attention was held more by attractive same-sex targets than attractive opposite-sex targets, *t* (23) = 3.018, *p* = 0.006, and average targets, *t* (23) = 2.520, *p* = 0.019. A 2 (priming condition) × 3 (target type) repeated measures ANOVA was conducted among single participants. There were a main effect for target type, *F* (2, 46) = 7.727, *p* = 0.001, partial η^2^ = 0.251, and a significant interaction for target type × priming condition, *F* (2, 46) = 5.345, *p* = 0.008, partial η^2^ = 0.189. Based on our interest, simple effects analyses revealed that love priming increased single females’ attention to attractive opposite-sex targets, *F* (1, 47) = 6.858, *p* = 0.012, partial η^2^ = 0.127. Paired-sample *t*-test showed that single females in the love-priming condition were more attentive to attractive opposite-sex targets than attractive same-sex targets, *t* (20) = 3.769, *p* = 0.001, and average targets, *t* (20) = 3.058, *p* = 0.006. For the love-priming condition, a 2 (relationship status) × 3 (target type) repeated measures ANOVA was conducted. There was a significant target type and relationship status interaction, *F* (2, 86) = 14.022, *p* < 0.001, partial η^2^ = 0.246. No other significant effects were observed (all *p*’s > 0.069). Simple effects analyses revealed that committed females were significantly less attentive than single females were to attractive opposite-sex targets, *F* (1, 43) = 10.732, *p* = 0.002, partial η^2^ = 0.200, and committed females were significantly more attentive than single females were to attractive same-sex targets, *F* (1, 43) = 6.851, *p* = 0.012, partial η^2^ = 0.137. There were no significant effects of relationship status, target type, or their interaction in the baseline condition (all *p*’s > 0.144).

The independent-samples *t*-test results confirmed that in the love-priming condition, the disengaging index on attractive same-sex targets was significantly greater than zero (zero indicates no attentional bias) among committed females, *t* (23) = 3.842, *p* = 0.013, indicating that committed females had difficulty in disengaging attention from attractive females. The disengaging index on attractive opposite-sex targets was also significantly greater than zero among single females, *t* (20) = 4.659, *p* = 0.006, indicating that single females had difficulty in disengaging attention from attractive males. The disengaging index on attractive opposite-sex targets was not significantly different from zero among committed females either in the baseline condition, *t* (34) = -0.457, *p* = 0.650, or in the love-priming condition, *t* (23) = -0.955, *p* = 0.349, indicating that committed females were inattentive to attractive males at the early stages of information processing, regardless of the condition they were allocated to. There were also no significant differences between zero and other disengaging indices (all *p*’s > 0.171).

**Fig 2:**
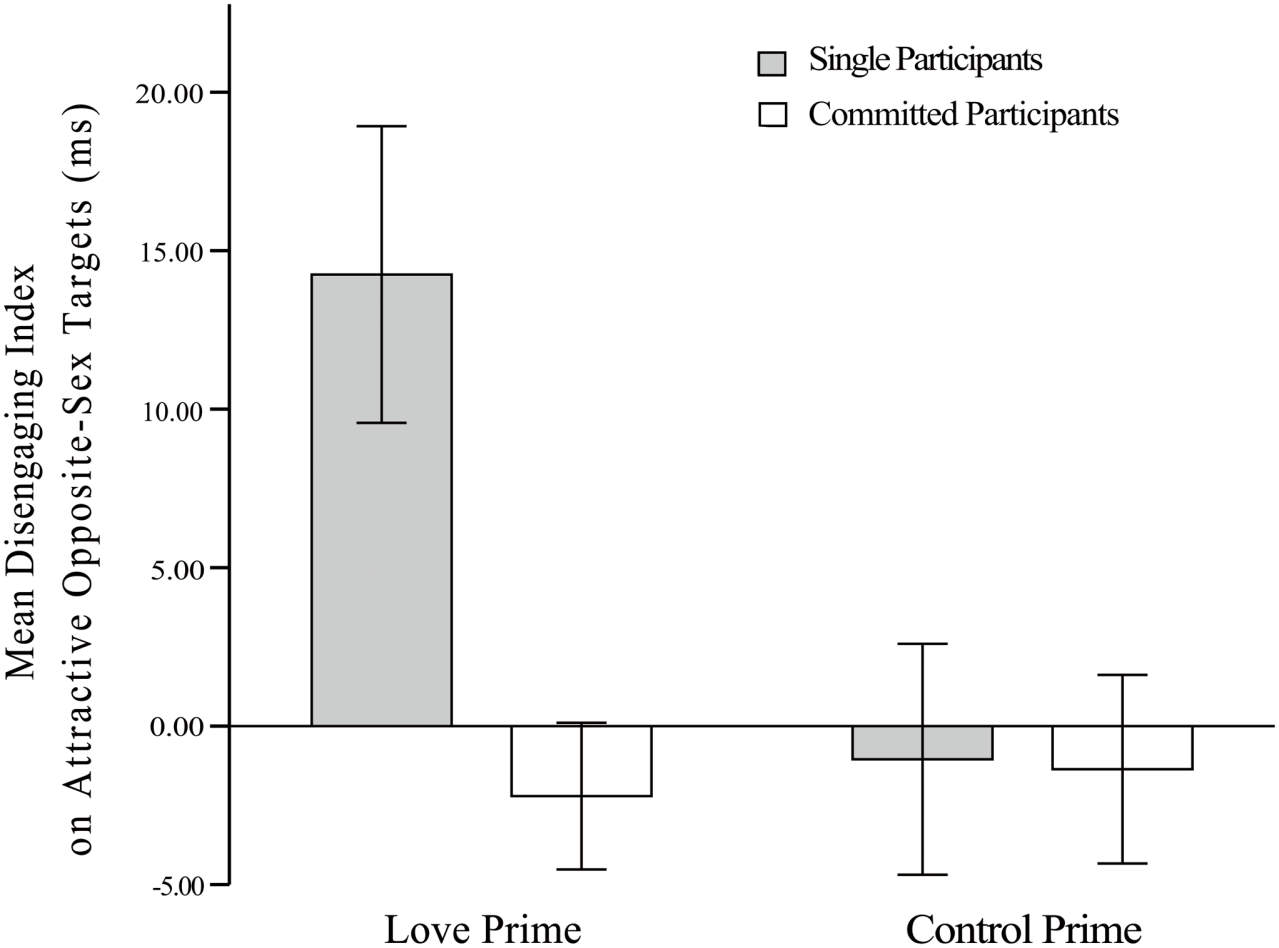
Mean disengaging index in relation to attractive opposite-sex targets. Note. Error bars show 1 SEM.

**Fig 3:**
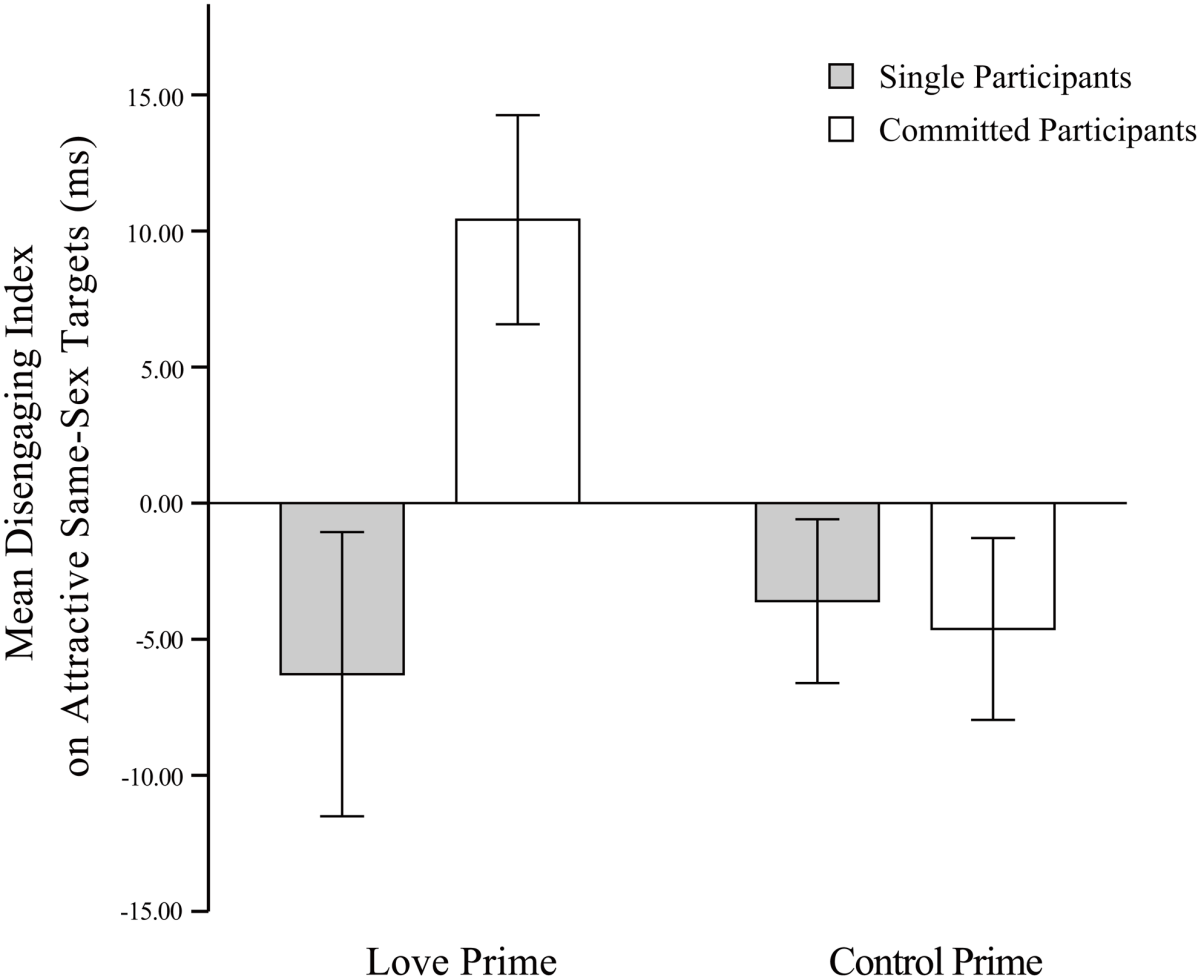
Mean disengaging index in relation to attractive same-sex targets. Note. Error bars show 1 SEM.

In summary, on the one hand, when romantic love was primed, single females showed increased attention to and difficulty in disengaging from attractive opposite-sex targets, but committed females were as inattentive to attractive opposite-sex targets as they were in the baseline condition (see Fig 2). On the other hand, committed females in the love-priming condition increased attention to and experienced difficulty with disengaging from attractive same-sex targets (see Fig 3).

### Engaging index

To test the engaging effects, a 2 (relationship status) × 2 (priming condition) × 3 (target type) repeated measures ANOVA was conducted for the engaging index. Null effects emerged for relationship status, *F* (1, 104) = 0.955, *p* = 0.331, partial η^2^ = 0.009; priming condition, *F* (1, 104) = 0.077, *p* = 0.782, partial η^2^ = 0.001; target type, *F* (2, 208) = 0.115, *p* = 0.892, partial η^2^ = 0.001; target type × priming condition, *F* (2, 208) = 1.342, *p* = 0.264, partial η^2^ = 0.013; target type × relationship status, *F* (2, 208) = 2.258, *p* = 0.107, partial η^2^ = 0.021; relationship status × priming condition, *F* (1, 104) = 0.062, *p* = 0.803, partial η^2^ = 0.001; or target type × relationship status × priming condition, *F* (2, 208) = 1.028, *p* = 0.360, partial η^2^ = 0.010. In addition, there were no significant differences between zero and any engaging indices (all *p*’s > 0.352).

## Discussion

To the best of our knowledge, this is the first study to explore the attentional bias associated with relationship maintenance among female college students in China. We used a modified dot-probe task that allowed for differentiation between the engage and disengage components of attention by adding neutral-neutral trials to explore the effects of different attention components for relationship maintenance purposes. Meanwhile, we calculated the engaging and disengaging indices by comparing RTs for face-neutral trials to neutral-neutral trials to control for overall group differences in RTs and to directly present participants’ attentional biases toward different facial stimuli under baseline and love-priming conditions. In the current study, we did not find significant engaging effects toward attractive faces among committed females. However, significant disengaging effects were found: in the love-priming condition, single females experienced difficulties with disengagement from attractive opposite-sex targets, but committed females remained inattentive to attractive males, much the same as they did in the baseline condition. In addition, committed females were inefficient at disengaging their attention from attractive same-sex targets when primed with romantic love.

Single females responded to love priming by increasing their attention toward attractive opposite-sex targets, but committed females did not. This suggests that love priming aroused interest toward attractive alternatives for single, but not for committed, females. Thus, our result might in part indicate that love acts as a commitment device that helps committed females disregard attractive alternatives, thus solidifying their commitment to the current relationship. In the current study, we added RTs of neutral-neutral pairs as one of the baseline measures and found that committed females were inattentive to attractive opposite-sex targets in both the baseline and love-priming conditions. This might fit with the Chinese cultural norm that women’s chastity is highly valued [21]. Evolutionary theories of mating suggest that women in long-term relationships tend to seek highly attractive mates from extrapair relationships who are good gene carriers [44]. However, in this study, Chinese females were inattentive to attractive alternatives at the early stage of perceptual processing, which may be a way of resisting temptation and remaining faithful to their ongoing relationship. However, in contrast to a previous study, in which it was found that committed individuals reduced their attention toward attractive alternatives when primed with romantic love [16], our study did not find the same priming effects. One possibility could be that the following methodological differences account for this discrepancy: (1) the study by Maner et al. [16] included both male and female participants, whereas there were only female participants in the current study. In addition, although the interaction between gender and priming effect did not approach significance in their study, the priming effect was somewhat larger among male participants (a reduction in attention of 134 ms) than among female participants (a reduction in attention of 40 ms). It seems that love priming had a weak effect for attractive opposite-sex targets among both Western and Chinese females. (2) In the current study, we used index scores to control for overall group differences in RTs, but Maner et al. [16] did not. In their study, committed participants’ RTs to the four target types in the love-priming condition were all less than their RTs in the control condition (see Figure 1 in their article). It seems reasonable to assume that the overall group differences in RTs amplified the priming effects for attractive opposite-sex targets among committed participants, especially females, in their study. Another possibility is that in view of the short presentation length of attractive opposite-sex faces, participants in the present study did not perceive the male targets as representing attractive alternatives that were available in reality. Thus, those attractive opposite-sex faces could not serve as strong threats to the relationship success of committed females [45]. Consequently, committed females in our sample did not show strong avoidance motivation toward those alternatives [46] (significant negative disengaging index) in either the baseline or love-priming conditions. However, committed women showed clear avoidance of the available attractive alternatives when the relationship motive was activated [47]. Further research should be conducted to examine the validity of this possibility by investigating attentional biases among committed Chinese females under different levels of relationship threat.

In the current study, committed females were significantly more inefficient in disengaging their attention from attractive same-sex targets when primed with romantic love than those in the baseline condition were. Some previous studies have suggested that Chinese students, especially female students, tend to associate love with jealousy and betrayal [30, 31]. Thus, being primed with love could easily trigger concerns about partner infidelity and relationship loss, which might activate a mate-guarding motive among the general population of committed Chinese females. Further, consistent with previous evidence [15, 18], the mate-guarding motive led committed individuals to hold their attention only on the attractive same-sex members. This fits with evolutionary theories suggesting that attractive women who can threaten one’s relationship and reproductive success serve as intrasexual rivals [48]. In addition, in China today, women’s status has improved enormously, but women are still subordinate to men within the marriage, which might lead to women paying higher costs for partners’ infidelity. Thus, attractive same-sex others may be considered a strong threat by committed Chinese females. The increased amounts of time spent dwelling on attractive females may help committed Chinese females identify and evaluate their potential rivals, and take action to protect their current relationship. However, Maner et al. [16] did not find significant priming effects on attractive same-sex targets, as we did in the current study. One possible explanation is that there are differences between Western and Eastern cultures in the ways in which romantic love is experienced. The Chinese view of romantic love stresses aspects related to sadness, jealousy, and betrayal, whereas Americans equate love with happiness [30, 49, 50]; as such, activation of the orbitofrontal cortex in the brain, which is associated with gains or losses, was found in Chinese, but not Western, college students who were currently in love. Chinese people may take negative aspects of romantic relationships into account more readily than Western participants do [31]. Thus, it seems that being primed with love could be more likely to trigger concerns about partner infidelity and relationship loss among the general Chinese population than in Western populations. Another possibility might be due to the different priming procedures employed. Maner et al. used scenario priming, asking participants to write a brief essay about a time in which they experienced strong feelings of love for their current partner [16]. This might have tended to elicit positive feelings associated with love. In the current study, we used semantic priming, which meant that the love words could activate positive or negative mental representations associated with the love of participants. In addition, as mentioned above, Chinese students tend to more readily associate love with negative features, so the love-priming procedure in the current study could easily triggered concerns about partner infidelity among our sample of Chinese participants. Further research should be conducted using the same love-priming procedure as Maner et al. [16], to examine the validity of this possibility.

In line with several studies of anxiety-related attentional bias [35, 51], our study did not observe engaging effects toward attractive individuals at the early stages of visual processing. One possible explanation is, as suggested by Fox et al. [51], that the initial orienting is an encapsulated process unaffected by the meaning or valence of the new object; however, biases in attentional disengagement facilitate the identification and evaluation of an implied threat. According to the findings from a study of anxiety-related attentional bias [33], another possibility is that attractive others might not be perceived as a strong threat by committed Chinese females, whereas a genuine threat would capture the attention of everyone [52]. A third possibility is the longer presentation of targets in this study. In previous studies, the engagement effect in regard to a threat has been obtained when shorter picture presentations (< 200 ms) were used [34]. The findings from our study do not exclude the possibility that facilitated engagement with a threat from an attractive other emerges at an earlier stage of information processing. This issue deserves further exploration in the area of relationship maintenance.

This research has limitations worth noting. One is that we did not explore the extent to which the different individual factors of observers shaped the early-stage information processing associated with relationship maintenance. For example, personality traits (e.g., avoidant attachment, high levels of chronic jealousy) might guide people’s selective attention toward attractive members [15, 53]. Feelings of not only romantic love, but also several other emotional states (e.g., jealousy or perceived threat) may influence observers’ attention toward attractive others [18, 43]. Applying specifically to women, the extent to which women attend to attractive opposite-sex targets may depend on their present stage of the menstrual cycle [54]. Future research should investigate the extent to which such factors influence people’s early-stage cognitive processing in the service of relationship maintenance. Another limitation is that the only target trait we chose to study was facial attractiveness. Other mating-related traits (e.g., waist-to-hip ratio, social dominance) carry different cues of reproductive value and may capture observers’ attention [55, 56]. Moreover, women tend to give more weight to social dominance than to physical attractiveness in mating-related evaluations of men [57]. Future research should investigate committed females’ relationship maintenance mechanisms in the conditions of different mating-related traits. The third limitation, already mentioned above, is that the method used in our study might not be sensitive to detecting engagement effects. Future research could use different methods, such as eye movement tracking, to assess biases in the specific component process of visual attention that might help to maintain a long-term romantic relationship.

## Conclusions

The current research adds new evidence from an Eastern cultural context to the existing theories on relationship maintenance. At the early stages of cognitive processing, committed Chinese females in this study protected an ongoing relationship by not only being inattentive to attractive males who could serve as attractive alternatives, but also being more attentive to attractive females who could serve as potential rivals when mental representations associated with romantic love were activated.

## Supporting Information

S1 Dataset SPSS data file showing raw RT data and related information for the participants. (ZIP)
